# The Abuse of Medical Charities

**Published:** 1873-10

**Authors:** 


### *"The Abuse of Medical Charities."*

The *N. Y. Medical Record* (Sept. 1, 1873,) contains an admirable editorial upon "The Abuse of Medical Charities," which concludes with the following: "When a profession can claim to treat gratuitously three hundred thousand paupers annually in New York City, not counting those in hospital, and knows at the same time that one out of every ten of the young medical men in that city can barely earn a living, we think it is about time to look into the matter, to consider whether the occasion is not opportune for proving the force of the proverb, 'Charity begins at home.'" We hope that every reader of the JOURNAL will read the entire article and make the application to his own locality, to which it is doubtless quite as appropriate as to that for which it was written. For the little consideration which the medical profession enjoys throughout this country, it is indebted to some of its own members, who, avaricious of practice and notoriety, have sought to acquire them by cheapening their own services, and thus taught society to fix a very low valuation upon that which is eagerly offered without fee or reward.

The world's standard of value is cost; that which costs little is, in the popular estimate, worth as little. At the present time the pay of the average physician is actually lower than that of the average mechanic, relatively very far below; and why? because the public knows it must pay for the services of the mechanic, whereas those of the physician can be obtained for nothing, or, at the most, for a promise to pay which is never meant to be redeemed, and of which the average physician has not the moral courage to enforce the fulfillment.

This cheapening of their profession works a double injury and perpetuates itself; first, by oppressing the young and obscure, whose services are esteemed valueless when the elders hold their own so cheap; and secondly, by thus discouraging the entrance into the profession of those who would maintain its honor and advance its interests. Thus the professional standard is actually approximated to that which it is made to appear, by some pseudo-philanthropists in its ranks.

The medical profession appears of late to have abandoned its *esprit de corps*, and its members to have concentrated and individualized their interests; instead of elevating the honor, dignity and importance of their profession and therewith their own as of integral parts of it, too many seem to be striving to rise alone in solitary grandeur upon its ruins.

There is one method of elevating the dignity of the profession, and of increasing the value of its services, which is within the reach of every physician; a method entirely within the comprehension of the public, and which it will most thoroughly appreciate, which if adopted and maintained consistently and persistently, will in one single year advance our profession in the public respect to a higher degree than can be accomplished in ten, by any other means. The method is this. Be strictly conscientious in rendering the best services within your capacity to your patients, and equally conscientious to yourselves in exacting a full equivalent for them, to be paid when the services are rendered; not at the end of the month, quarter, or year, but on the spot. In other words, treat your patient as he treats you. Let us ask no protection for our profession, no privileges, but simply the privilege to protect ourselves.

When you are asked for charity you will then have the wherewithal to do charity, you will be able to give out of that which you have received. In plain English, adopt the cash system as the rule of compensation for professional services, and we can assure those who will try it for one year, that they will never abandon it. *Experto crede.* H.

The *New York Medical Journal*, for September, 1873, announces that "Dr. Walter Hay, of Chicago, has been appointed Adjunct Professor of Principles and Practice of Medicine in the Western Medical Institution." This is news indeed to the one most interested. We had the honor to be appointed to that position in Rush Medical College, but "the Western Medical Institution " is something which we know not of. Will the *New York Medical Journal* please relieve us of the unmerited honor and oblige H.

